# Isotopic niche overlap among foraging marine turtle species in the Gulf of Mexico

**DOI:** 10.1002/ece3.10741

**Published:** 2023-11-28

**Authors:** Savannah Weber, Joshua A. Cullen, Mariana M. P. B. Fuentes

**Affiliations:** ^1^ Department of Earth, Ocean, and Atmospheric Science Florida State University Tallahassee Florida USA

**Keywords:** foraging ecology, marine turtles, niche overlap, stable isotope, sulfur isotope, sympatric species

## Abstract

Sympatric species may overlap in their use of habitat and dietary resources, which can increase competition. Comparing the ecological niches and quantifying the degree of niche overlap among these species can provide insights into the extent of resource overlap. This information can be used to guide multispecies management approaches tailored to protect priority habitats that offer the most resources for multiple species. Stable isotope analysis is a valuable tool used to investigate spatial and trophic niches, though few studies have employed this method for comparisons among sympatric marine turtle species. For this study, stable carbon, nitrogen, and sulfur isotope values from epidermis tissue were used to quantify isotopic overlap and compare isotopic niche size in loggerhead (*Caretta caretta*), green (*Chelonia mydas*), and Kemp's ridley (*Lepidochelys kempii*) turtles sampled from a shared foraging area located offshore of Crystal River, Florida, USA. Overall, the results revealed high degrees of isotopic overlap (>68%) among species, particularly between loggerhead and Kemp's ridley turtles (85 to 91%), which indicates there may be interspecific competition for resources. Samples from green turtles had the widest range of isotopic values, indicating they exhibit higher variability in diet and habitat type. Samples from loggerhead turtles had the most enriched mean δ^34^S, suggesting they may forage in slightly different micro‐environments compared with the other species. Finally, samples from Kemp's ridley turtles exhibited the smallest niche size, which is indicative of a narrower use of resources. This is one of the first studies to investigate resource use in a multispecies foraging aggregation of marine turtles using three isotopic tracers. These findings provide a foundation for future research into the foraging ecology of sympatric marine turtle species and can be used to inform effective multispecies management efforts.

## Graphical abstract

Understanding how sympatric species compare in resource use can provide insight into their ecological role and aspects of their foraging ecology. To investigate this, we collected skin samples for carbon, nitrogen, and sulfur stable isotope analysis from loggerhead, green, and Kemp's ridley turtles sampled in a shared coastal foraging area located in the northeastern Gulf of Mexico. Our results revealed high degrees of isotopic overlap among all three species, which indicates little resource partitioning and further suggests that there may be interspecific competition for resources.

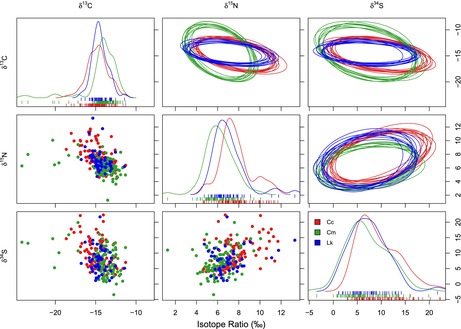

## 1 INTRODUCTION

Patterns of resource use by a species can have significant effects on the local ecosystem by influencing interactions within a community, the dynamics of resource availability, and the overall distribution and abundance of organisms (Chesson, 2000; Ross, 1986; Sale, 1974). Thus, an understanding of how species use resources can provide insights into their ecological role and aspects of their foraging ecology, which may be used to identify key foraging areas (Devictor et al., 2010; Gama et al., 2021; Kent et al., 2017). The latter is important as it can help determine priority habitat for conservation based on areas that offer the most dietary resources for threatened and endangered species (Lamont & Iverson, 2018; Oksanen et al., 2015). The resources used by species can be estimated by characterizing their ecological niche, which represents the entirety of an organism's interactions within its biotic and abiotic environment (Hutchinson, 1978; McGill et al., 2006).

Determining resource use among different species that overlap in habitat (i.e., sympatric species) can provide insights into differences between their spatial and trophic ecologies (Borrell et al., 2021). Sympatric species are typically thought to compete for local resources (Connell, 1961) and often exhibit resource partitioning to reduce competition (Chase & Leibold, 2003; MacArthur & Levins, 1964). Measuring niche overlap (i.e., resource overlap) along dimensions such as food source and habitat type can indicate the degree to which co‐occurring species are dividing or sharing resources and help determine the mechanisms of species coexistence (HilleRisLambers et al., 2012; Hutchinson, 1961; Sale, 1974). Additionally, information on niche overlap may be used to guide multispecies management approaches by quantifying the ecological similarities between species, which can facilitate the efficient use of financial efforts to aid in the protection of habitat and dietary resources shared by multiple species (Elafri et al., 2017; Laub & Budy, 2015; Monda & Ratti, 1988).

Despite the importance of understanding resource use among sympatric species, there is limited research on this topic for marine turtles (Lamont & Iverson, 2018; Melo‐Merino et al., 2020). Marine turtle species forage across different trophic levels, with species often co‐occurring in the same foraging habitats (Haywood et al., 2019). Previous studies have used a variety of methods to characterize specific aspects of coexisting marine turtle species' ecological niches. Satellite telemetry coupled with ecological niche modeling has been used to investigate spatial niches (DiMatteo et al., 2022; Fujisaki et al., 2020; Hart et al., 2018), and dietary analyses via gastric lavages (Martins et al., 2020) or gut content analysis (Palmer et al., 2021; Stringell et al., 2016) have been employed to characterize trophic niches. However, these approaches can be limiting since they portray only the spatial niche (i.e., habitat use) or the trophic niche (i.e., diet) of a species. Stable isotope analysis (SIA) can be used instead to characterize the species' isotopic niche, which represents both the spatial and trophic niches, and thus provides a proxy of the ecological niche (Newsome et al., 2007; Vander Zanden et al., 2013). Typically, carbon (δ^13^C) and nitrogen (δ^15^N) bulk isotope ratios are used to characterize marine turtle niches (Haywood et al., 2019). The δ^13^C value of the consumer is indicative of habitat type and primary carbon source (García‐Vernet et al., 2021), while δ^15^N can be used to indicate the trophic position of the consumer since δ^15^N experiences a substantial amount of enrichment with each step up the food web (Bradshaw et al., 2017; Hussey et al., 2014; Rossman et al., 2016). Most of the studies to date that use bulk isotope ratios to characterize marine turtle niches focus on carbon (δ^13^C) and nitrogen (δ^15^N) (Haywood et al., 2019). However, the addition of a third isotopic marker, sulfur (δ^34^S), can provide additional insights since δ^34^S isotope ratios exhibit limited trophic fractionation and can be used to determine differences between benthic and pelagic productivity pathways in coastal systems (Chan et al., 2022; García‐Vernet et al., 2021; Peterson & Fry, 1987). This is because primary producers that use different sources of sulfur will have different δ^34^S values (Connolly et al., 2004). For example, producers such as microalgae and phytoplankton mainly use seawater sulfates that are enriched in ^34^S, while benthic algae and rooted plants primarily use sedimentary sulfides that are more depleted in ^34^S (Borrell et al., 2021; Connolly et al., 2004).

Most of the marine turtle SIA studies to date have focused on a single marine turtle species (Figgener et al., 2019; Haywood et al., 2019), with only a few studies investigating the spatial and trophic ecologies of multiple species. These studies compared δ^13^C and δ^15^N values from different species of nesting females (Filippos et al., 2021), recently recruited and oceanic stage juveniles (Reich et al., 2007), and stranded turtles (Godley et al., 1998). Only one SIA study to date has investigated resource use among a multispecies foraging aggregation, which quantified the isotopic overlap between green turtles (*Chelonia mydas*) and hawksbill turtles (*Eretmochelys imbricata*) (Clyde‐Brockway et al., 2022). The lack of multispecies SIA studies clearly highlights a gap in knowledge as to how other species of marine turtles are using resources within the same foraging area.

To address this research gap and further improve our understanding of resource use among sympatric species of marine turtles, the stable isotope values for δ^13^C, δ^15^N, and δ^34^S were used to characterize the isotopic niches of loggerhead turtles (*Caretta caretta*), green turtles, and Kemp's ridley turtles (*Lepidochelys kempii*) from a foraging area located off the coast of Crystal River, Florida, USA within the northeastern Gulf of Mexico (GoM). Generally, green turtles shift from an omnivorous to herbivorous diet when they migrate to neritic foraging habitats, while loggerhead and Kemp's ridley turtles feed on benthic invertebrates (Valverde & Holzwart, 2017). More specifically, the aims of this study were to 1) compare niche volume and position among species, 2) calculate niche overlap between pairs of species, and 3) evaluate the spatial and foraging ecology of each species. These three species of marine turtles exhibit multi‐year fidelity to foraging habitat within the northeastern GoM region (Barichivich, 2006; Chabot et al., 2021; Schmid et al., 2003; Wildermann et al., 2019) and likely overlap in both habitat and dietary resource use (Lamont & Iverson, 2018; Lamont & Johnson, 2021), providing an ideal system to explore niche overlap among marine turtle species and characterize their foraging ecologies.

## 2 METHODS

### 2.1 Study site and sample collection

**FIGURE 1:**
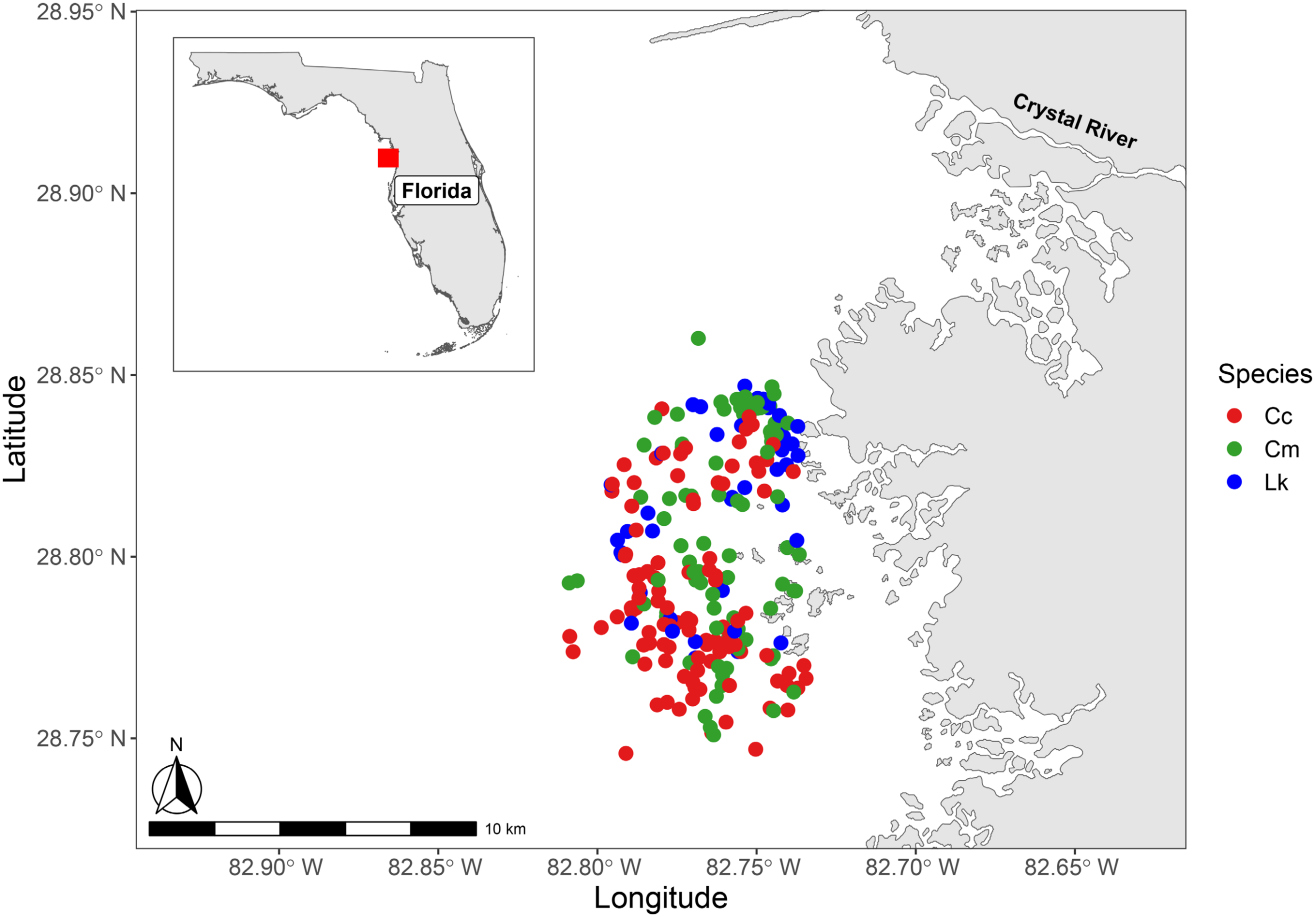
Map of study site and capture locations for loggerhead (Cc), green (Cm), and Kemp's ridley (Lk) turtles sampled off the coast of Crystal River, Florida, USA between 2016 and 2022.

The neritic waters off the coast of Crystal River, Florida, USA (Figure 1) have been identified as an important foraging area for juvenile green and Kemp's ridley turtles, as well as subadult to adult loggerheads (Chabot et al., 2021; Wildermann et al., 2019, 2020). As part of a long‐term in‐water monitoring program to determine marine turtle population structure in the region, strip transects were conducted between 2016 and 2022. Turtle sightings were recorded opportunistically using a GPS Garmin 62 s, and turtles were captured by dipnet or the rodeo technique (Fuentes et al., 2006; Limpus & Walter, 1980) and brought to the boat to be processed. A passive integrated transponder (PIT tag, Biomark, GPT 12) and Inconel flipper tags (Style 681 National Band and Tag Company, Newport, USA) were applied, when not already present, following protocols from the Florida Fish and Wildlife Conservation Commission Marine Turtle Conservation Handbook (FFWCC, 2016). Captured turtles were placed in a protected area on the deck of the boat and body measurements (±0.1 cm) were recorded using a tape measure and calipers to measure curved carapace length and straight carapace length, respectively. The minimum curved carapace length (hereafter referred to as CCL), defined as the length between the nuchal notch and caudal notch (Bolten, 1999), was used to characterize the life stages of each species. Epidermis samples for SIA were collected from the shoulder of each turtle using a 5 mm biopsy punch and were then stored in a vial with salt until analysis (as per Silver‐Gorges et al., 2021).

Epidermis samples were cleaned using deionized water to remove salt and particulate matter, and the epidermis was separated from the underlying dermis tissue and diced using a sterile scalpel. The samples were then oven‐dried at 60°C for 48 h or freeze‐dried at −50°C for 12 h to remove moisture. Due to technical and personnel limitations, stable isotope analyses were then carried out at three laboratories: Fish and Wildlife Research Institute of the Florida Fish and Wildlife Conservation Commission and Marine Environmental Chemistry Laboratory at the University of South Florida College of Marine Science (FWRI USF), Marine Biological Laboratory (MBL) Stable Isotope Laboratory (Woods Hole, Massachusetts), and Washington State University (WSU) Stable Isotope Core Laboratory (see Table S1 for further details).

For carbon and nitrogen isotope analysis, an accelerated solvent extractor (Model 200, Dionex) was used to extract lipids from the samples using petroleum ether (three cycles of 5 min heating followed by 5 min static purging). Samples were weighed to 0.5–0.7 mg using a Mettler Toledo microbalance and then placed into Costech tin cups and converted into N_2_ and CO_2_ via combustion using a Carlo‐Erba NA2500 Series 2 Elemental Analyzer (Thermoquest Italia). For sulfur isotope analysis, each sample consisted of 3 mg bulk tissue from each turtle, which was loaded into sterilized tin capsules and then combusted with an elemental analyzer (ECS 4010; Costech Analytical). The SO_2_ gases were then separated with a 0.8 mg GC column at 100°C.

For samples sent to FWRI USF and WSU, isotope ratios were measured in a continuous‐flow mass spectrometer (Delta Plus XP, ThermoFinnigan). For samples sent to MBL, isotope ratios were measured using a Europa20‐20 continuous‐flow isotope ratio mass spectrometer interfaced with a Europa ANCA‐SL elemental analyzer. The stable isotope values are expressed in δ notation as per mil (‰) according to the following equation:

$$\delta X=\left[\left(R_{\text{sample}}/R_{\text{standard}}\right)-1\right]\times 1000$$

where *X* is ^13^C, ^15^N, or ^34^S and R_sample_ is the ratio of ^13^C:^12^C, ^15^N:^14^N, or ^34^S:^32^S in the tissue sample. The reference material (*R*_standard_) used for 13C and 15N is relative to the international standards of Vienna PeeDee Belemnite and atmospheric nitrogen, respectively. Reference material for 34S consisted of IAEA‐S‐1 silver sulfide, and sulfur isotope ratios were reported per mil (‰) relative to Vienna Canyon Diablo Troilite. Estimates of analytical precision are reported in Table S1. Although there may be isotopic variation among values from the different accredited laboratories, this is not expected to have a significant impact on the results as potential variability among labs is usually less than 0.5‰ (Ceriani et al., 2014).

### 2.2 Data analyses

Ranges, means, and standard deviations (SDs) for CCL, δ^13^C, δ^15^N, and δ^34^S were calculated for each species using R v4.1.3 (R Core Team, 2021). The hypervolume niche size and niche overlap among species were calculated using the R package “nicheROVER” (Lysy et al., 2014). “nicheROVER” uses a Bayesian framework to quantify probabilistic metrics in niche space and is not restricted to two dimensions (Swanson et al., 2015). For each Bayesian model, 10,000 Markov chain Monte Carlo (MCMC) iterations were used to characterize the posterior distributions for isotope values of each species (mean and variance–covariance matrix) using an uninformative Normal‐Inverse‐Wishart prior (Lysy et al., 2014). Niche size was defined as the species niche region with a 95% probability of finding a specific individual of that particular species (García‐Vernet et al., 2021), and this was estimated by calculating a point estimate of the mean niche size across posterior sample of mean μ and covariance ∑ (Swanson et al., 2015). The parameter μ is a vector of length 3 (i.e., the number of isotopes) that stores the mean isotope values for a given species, while ∑ is a 3 × 3 matrix of the variances and covariances of the isotopes for a particular species that characterizes the shape of the niche hypervolume.

Niche overlap was defined as the percent probability of an individual from one species falling within the niche space of another species, and thus was estimated as the overlap in hypervolume niche space (Borrell et al., 2021; Swanson et al., 2015). Uncertainty in niche overlap for each species of turtle and each isotope pair was reported as the posterior distribution of the overlap percentage, and Bayesian 95% credible intervals for each pairwise comparison were calculated (Borrell et al., 2021). To calculate overlap, the alpha value was set as 0.95, as this provides the 95% probability region of the three‐dimensional isotopic niche (as per Swanson et al., 2015). A 95% probability ellipse is considered to be a more accurate measurement of overlap than the commonly used 40% probability ellipse used for bivariate Standard Ellipse Areas (García‐Vernet et al., 2021, see Jackson et al., 2011).

## 3 RESULTS

Epidermis samples were obtained from 104 loggerhead turtles, 95 green turtles, and 49 Kemp's ridley turtles that were captured opportunistically from 2016 to 2022. Body size ranged from 53.6 to 105.8 cm CCL (mean ± SD: 80.0 ± 12.6) for loggerhead turtles, 26.0 to 73.4 cm CCL (mean ± SD: 40.1 ± 7.99) for green turtles, and 26.4 to 61.0 cm CCL (mean ± SD: 46.7 ± 8.36) for Kemp's ridley turtles. Based on these values, the loggerhead turtles were categorized as subadults and adults (Benscoter et al., 2022; Bjorndal et al., 2001), and the green and Kemp's ridley turtles were categorized as juveniles (Eaton et al., 2008).

**FIGURE 2:**
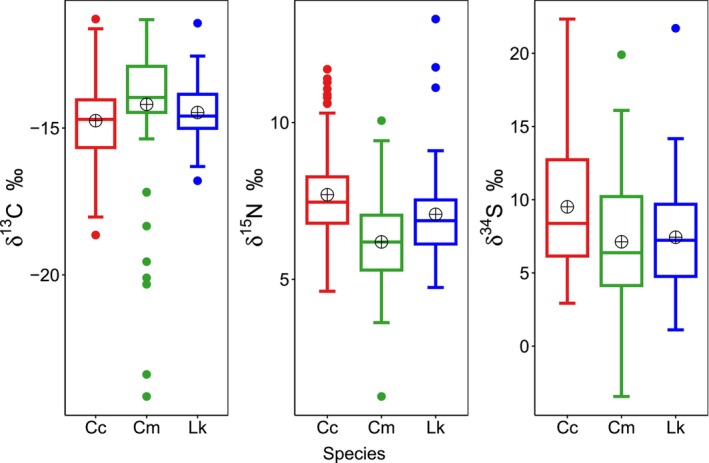
Box plots displaying the distribution of the δ^13^C, δ^15^N, and δ^34^S isotope data generated from samples taken from loggerhead (Cc), green (Cm), and Kemp's ridley (Lk) turtles off the coast of Crystal River, Florida, USA, between 2016 and 2022. The median is represented as the horizontal line, the mean is displayed as the crossed circle, and the outliers are shown as points. The gray boxes extend to the 25th and 75th percentile, while the lower and upper whiskers extend from the 10th to the 90th percentile.

**FIGURE 3:**
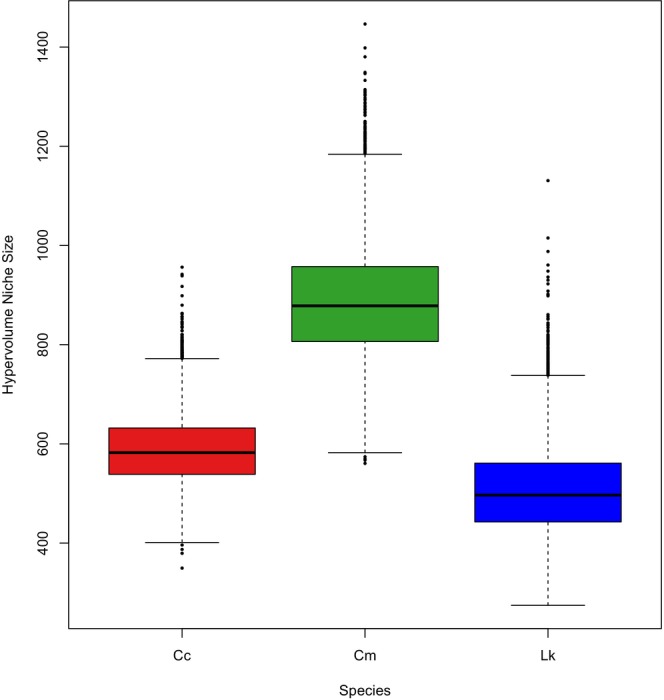
Boxplot showing the distribution of the estimated (95% probability) isotopic niche hypervolume (‰^3^) based on the posterior distributions for loggerhead (Cc), green (Cm), and Kemp's ridley (Lk) turtles. The medians are represented by the solid horizontal lines, inter‐quartile ranges by the boxes, whiskers by the 10th to the 90th percentile, and outliers by the black points.

Mean isotopic values of the three species ranged from −14.7 to −14.2‰ for δ^13^C, 6.19 to 7.70‰ for δ^15^N, and 7.13 to 9.51‰ for δ^34^S (Table 1). Samples from all three species had similar δ^13^C means, with only a 0.5‰ difference between the most enriched (green: −14.2‰) and most depleted (loggerhead: −14.7‰) mean values (Table 1). For δ^15^N and δ^34^S, samples from loggerhead turtles were the most enriched, while samples from green turtles were the most depleted (Figure 2). The loggerhead and green samples had a 1.5‰ difference between δ^15^N mean values and a 2.4‰ difference between δ^34^S mean values (Table 1). Additionally, samples from loggerhead turtles exhibited the narrowest isotopic ranges for δ^15^N and δ^34^S, while samples from Kemp's ridley turtles presented the narrowest range for δ^13^C (Table 1). Samples from green turtles had the widest range in isotopic values for δ^13^C, δ^15^N, and δ^34^S (Table 1), and therefore, the largest estimated niche size (mean [95% CI]: 886.31 ‰^3^ [689.07, 1129.33]) (Figure 3). The niche size of green turtles was approximately 1.5 times larger than that of loggerhead turtles (mean [95% CI]: 587.95 ‰^3^ [463.07, 744.24]) and 1.7 times larger than that of Kemp's ridley turtles (mean [95% CI]: 506.62 ‰^3^ [357.34, 704.05]) (Figure 3). These niche size estimates were further supported by the probabilistic pairwise niche size comparisons, which showed that green turtles were > 99% more likely to occupy a larger niche than loggerhead and Kemp's ridley turtles (Table 2). Conversely, Kemp's ridley turtles were 22.7% and 0.47% more likely to occupy a larger niche than loggerhead and green turtles, respectively (Table 2). This means there is a higher probability that the niche size of Kemp's ridley turtles is smaller than loggerhead and green turtles.

**TABLE 1:** Minimum and maximum values, ΔRanges (represented here as difference between maximum and minimum value), means, and standard deviations of δ^13^C, δ^15^N, and δ^34^S values for samples from loggerhead, green, and Kemp's ridley turtles off the coast of Crystal River, Florida, USA between 2016 and 2022.

	δ^13^C (‰)			δ^15^N (‰)			δ^34^S (‰)		
	Min, max	Δ Range	Mean ± SD	Min, max	Δ Range	Mean ± SD	Min, max	Δ Range	Mean ± SD
Loggerhead, *n* = 104	−18.6, −11.3	7.3	−14.7 ± 1.32	4.62, 11.7	7.1	7.7 ± 1.52	2.93, 22.3	19.4	9.5 ± 4.35
Green, *n* = 95	−24.1, −11.3	12.8	−14.2 ± 2.10	1.26, 10.1	8.8	6.2 ± 1.31	−3.44, 19.9	23.3	7.1 ± 4.27
Kemp's ridley, *n* = 49	−16.8, −11.4	5.4	−14.5 ± 0.98	4.74, 13.3	8.6	7.1 ± 1.62	1.11, 21.7	20.6	7.4 ± 4.00

**TABLE 2:** Estimated probability that niche hypervolume size of one species was greater than another.

>	Loggerhead	Green	Kemp's ridley
Loggerhead	–	0.91%	77.3%
Green	99.1%	–	99.5%
Kemp's ridley	22.7%	0.47%	–

*Note*: The table is to be read across each row, for example, there was a 99.1% probability of green turtle niche size being greater than that of loggerhead turtles, and therefore a 0.91% probability that the niche size of loggerhead turtles was greater than green turtles.

**FIGURE 4:**
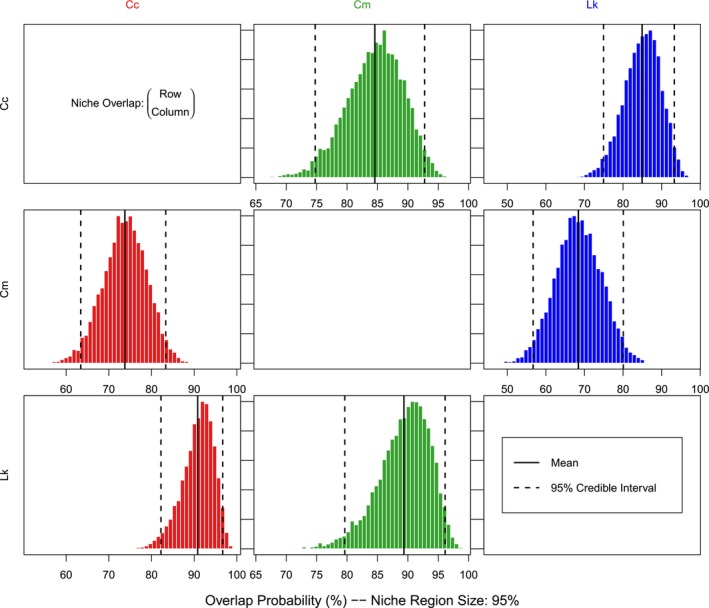
Posterior distribution of the probabilistic niche overlap metric (%) between loggerhead (Cc), green (Cm), and Kemp's ridley (Lk) turtles in Crystal River, Florida, calculated using the hypervolume niche space using nicheROVER. Overlap is interpreted as the probability that the species in the row was found in the niche of the species in the column (e.g., There is a 73.80% probability that Cm was found within the niche of Cc). The dashed lines represent 95% credible intervals, and the solid black line displays the mean.

**FIGURE 5:**
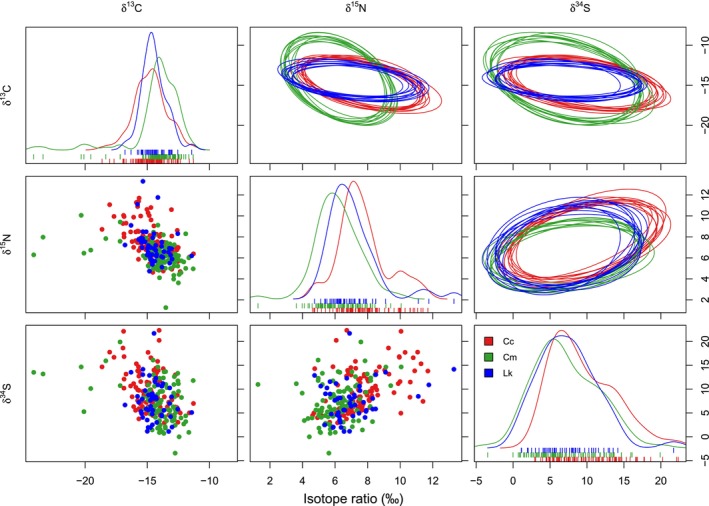
NicheROVER plots for loggerhead (Cc), green (Cm), and Kemp's ridley (Lk) turtles sampled off the coast of Crystal River, Florida, USA between 2016 and 2022. Top‐right: Ten random samples of two‐dimensional ellipses from the posterior distribution (95% probability region) for each pair of isotope ratios. Diagonal: One‐dimensional density plots showing distribution of isotope values with rug plots to show individual values. Bottom‐left: Scatterplots of raw data for each pair of isotopes.

A substantial amount of niche overlap (>68%) was found among the isotopic hypervolumes of all three species (Figure 4), which was also supported by visualizing niche ellipses on two‐dimensional isotopic biplots (Figure 5). Kemp's ridley turtles had the highest probability of being found within the niche regions of the other turtle species, with a 90.88% probability of occurrence within the loggerhead turtle niche and an 89.30% probability of occurrence within the green turtle niche (Figure 4). Green turtles exhibited the lowest probability of overlap between the other species' niche regions, with a 73.80% probability of occurrence within the loggerhead turtle niche and a 68.34% likelihood of occurrence within the Kemp's ridley turtle niche (Figure 4). For the loggerhead turtles, there was an 84.65% chance of being found within the green turtle niche, and an 85.03% chance of being found within the Kemp's ridley turtle niche (Figure 4). Thus, the highest percentage of overlap between all species' niche regions was between Kemp's ridley and loggerhead turtles (Figures 4 and 5).

## 4 DISCUSSION

The present study is one of the first to use SIA to assess resource use within a foraging aggregation of multiple species of marine turtles. The overall isotopic means and ranges were similar among subadult and adult loggerhead, juvenile green, and juvenile Kemp's ridley turtles, which suggests little resource partitioning in general foraging area and diet among these consumer species. Samples from all three species exhibited similar δ^13^C values, indicating they occupy the same general foraging environment, as is expected for sympatric species. Additionally, the similar δ^13^C values suggest that they forage on prey items that are likely from similar benthic‐based food webs (Plotkin et al., 1993; Williams et al., 2014; Witzell & Schmid, 2005). Mean δ^15^N values were also similar between samples of loggerhead and Kemp's ridley turtles (Table 1), suggesting these species likely forage at similar trophic levels. Samples from green turtles had a slightly depleted mean δ^15^N value (Range: 1.26–10.1‰; Mean: 6.2 ± 1.31‰) compared with the other two species (Kemp's ridley: Range: 4.74–13.3‰; Mean: 7.1 ± 1.62‰; loggerhead: Range: 4.62–11.7‰; Mean: 7.7 ± 1.52‰), which likely indicates more of an herbivorous diet comprised of seagrass and macroalgae (Bjorndal, 1985). However, it is worth noting that while the δ^15^N values differed among species, there may not be a substantial ecological significance as the observed maximum difference (1.5‰) did not exceed the general discrimination factor of 3.4‰ for δ^15^N (Post, 2002, but see Hussey et al., 2014). Samples from green turtles also had the widest ranges for δ^13^C, δ^15^N, and δ^34^S (Table 1), which may suggest that they use a wider range of resources and exhibit higher variability in diet and habitat type, and thus may be more generalists (Chan et al., 2022). This is further supported by their larger niche size estimate compared with the other species. Indeed, samples from loggerhead turtles had the narrowest range for δ^15^N and δ^34^S and Kemp's ridley turtles occupied the smallest niche size. This suggests that these two species may exhibit stronger habitat and dietary preferences compared with juvenile green turtles. This likely results from juvenile green turtles undergoing an ontogenetic shift in habitat from pelagic to benthic resources (Arthur et al., 2008). Once they recruit to coastal foraging habitats, they forage primarily on aquatic vegetation (e.g., seagrass, benthic macroalgae) while still feeding on small amounts of invertebrates, whereas loggerhead and Kemp's ridley turtles primarily feed on benthic invertebrates (Valverde & Holzwart, 2017). This ontogenetic shift could be a major source of the observed isotopic variation contributing to the wider isotopic niche of green turtles (Figure S1). It is possible that within the juvenile green turtle population, there may be size‐related differences in resource use, as has been found in the loggerhead (Silver‐Gorges et al., 2023) and Kemp's ridley (Weber et al., 2023) turtle populations within the Crystal River foraging site. Although turtle size‐partitioning was beyond the scope of this study, we plotted SIA values against marine turtle size (Figure S2). Future efforts could determine SIA values over time using scute tissue to investigate the potential relationship with size among green turtles in the Crystal River population (e.g., Cardona et al., 2010).

The narrower range and enriched mean δ^34^S value from loggerhead turtle samples suggest that their prey may have less benthic and coastal influence than the other species (Guillemin et al., 2022). Specifically, the narrower range of δ^34^S indicates that loggerheads may exhibit specialization for micro‐environments that have a different primary sulfur source. However, these micro‐environments are likely still located within the overall foraging area of green and Kemp's ridley turtles, as all three species exhibit similar δ^13^C values (Sullivan & Moncreiff, 1990). The more enriched δ^34^S values could indicate that loggerhead turtles forage slightly farther from the coast, or in deeper waters compared with the other two species (Borrell et al., 2021). This was observed over broad spatial scales in the GoM through satellite telemetry, where loggerhead turtles were found to be foraging in deeper waters and farther from shore than Kemp's ridley turtles (Hart et al., 2018). Similar spatial partitioning was found between Kemp's ridley and loggerhead turtles in Chesapeake Bay, as food preferences drove Kemp's ridleys to occupy shallower areas, while loggerheads occupied deeper areas (DiMatteo et al., 2022). Indeed, a previous study using satellite tracking information from marine turtles at the Crystal River study site indicated that green and Kemp's ridley turtles occupied more nearshore areas (<5 km from mainland), while the core area of loggerhead turtles was found to be further from the shore (5–10 km from mainland) (see Wildermann et al., 2019). Future studies should aim to obtain further fine‐scale information on habitat partitioning across species and among foraging aggregations by coupling SIA with acoustic or satellite telemetry (e.g., Fastloc GPS tags) (Lamont & Iverson, 2018).

Previous studies within the northeastern Gulf of Mexico (GoM) have shown that all three species exhibit multi‐year site fidelity to their foraging grounds (Conant et al., 2009; Lamont & Johnson, 2021; Tucker et al., 2014). Since marine turtle epidermis tissue has a 4–8‐month turnover rate, thus representing the diet and habitat use from several months prior (Reich et al., 2008; Tucker et al., 2014), the similar δ^13^C values among individuals in this study suggest that the Crystal River foraging aggregation may also exhibit long‐term site fidelity. However, it is possible that some individuals could be migrants from different regions that have similar environmental conditions, resulting in these individuals exhibiting similar isotopic values to those that are residents in Crystal River. Given that the Crystal River foraging habitat is centered on a gradient in baseline isotope values along the West Florida Shelf (Radabaugh et al., 2013), it is also possible that this area may contain a blend of isotopic signatures across different geographic regions, resulting in the similar isotopic values among the turtle species. Additionally, there are potential outliers present, particularly from several green turtles that exhibited more depleted δ^13^C values (Figure 2). The CCL from these 8 individuals ranged from 26.0 to 45.0 cm (mean ± SD: 37.4 ± 4.6), though other individuals within this size range exhibited δ^13^C values that were similar to the overall mean (Figure S1). The general range of size of recruitment to neritic developmental habitats for green turtles is 30–40 cm CCL (Bjorndal & Bolten, 1995), so it is possible that these outlier values could be from new recruits exhibiting more depleted δ^13^C values reflective of their previous pelagic and offshore habitat, or they could be from migrating individuals (see Figure S3, for analysis with omission of these outliers). It is also worth noting that these outlier turtles were captured across different seasons and years, and thus seasonal/annual variation did not influence the observed δ^13^C values.

The Bayesian niche models indicated that there was a substantial degree of niche overlap among all three species (>68%), which suggests that there may be interspecific competition for habitat and dietary resources within the marine turtle populations in Crystal River (Borrell et al., 2021). However, these inferences should be made with caution since isotopic overlap does not directly reflect overlap in diet among species (Stewart et al., 2017). Factors such as resource availability, relative abundance of each species, and variations in nutrient cycling can contribute to the observed overlap (Swanson et al., 2015). Additionally, the different turtle species could have dietary preferences for prey items that are isotopically similar, but taxonomically distinct, resulting in similar isotopic values (Chan et al., 2022; Stewart et al., 2017). It is also possible that there are ample dietary resources that adequately support the number of turtles within the study area, and thus, there may not be any competition for resources. Further investigations into dietary differences among species could include the applications of additional biotracers, such as compound‐specific isotopic analyses of amino acids (CSIA‐AA), and heavy metal analysis (Gardner et al., 2006; Seminoff et al., 2021).

It is generally believed that these three marine turtle species can coexist in the same foraging area because each species is specialized to a different type of diet (Lamont et al., 2022). Previous dietary analyses have shown that in the northeastern GoM, green turtles primarily consume seagrass and Kemp's ridley turtles primarily consume crabs (Barichivich et al., 1999; Foley et al., 2007). No direct dietary studies have been conducted for loggerhead turtles in the northeastern GoM, though a study in the northwestern GoM (i.e., Texas) found that they primarily consume sea pens, crabs, and mollusks (Plotkin et al., 1993). However, additional dietary studies within the northeastern GoM have reported other major prey items that could overlap among the species in the present study. Green turtles have been reported to forage on tunicates (Herren, 2018; Williams et al., 2014), as have Kemp's ridley turtles (Witzell & Schmid, 2005). Kemp's ridley turtles have been found to also forage on less common prey items, such as fish and horseshoe crabs (Servis et al., 2015). This suggests that Kemp's ridley turtles may exhibit opportunistic feeding preferences and select easily captured prey when encountered (Witzell & Schmid, 2005). Another possibility is that Kemp's ridleys' preference for these less common prey types could be a response of resource partitioning with loggerhead turtles, as they may be competing for crab prey species (Servis et al., 2015). Loggerhead turtles in the northeastern GoM could have similar dietary preferences as their conspecifics in the northwestern GoM and thus select for crab species, therefore overlapping their diet with Kemp's ridley turtles. Indeed, comparisons of fecal analysis from loggerhead and Kemp's ridley turtles in a foraging area in Long Island, New York, found substantial dietary overlap as both species predominantly fed on spider crabs and rock crabs (Burke et al., 1993). Overall, the findings from previous studies suggest that there could be dietary overlap of tunicate prey between Kemp's ridley and green turtles, and crab prey between Kemp's ridley and loggerhead turtles. This could explain why Kemp's ridley turtles had the highest probability of being found within the niche regions of the other turtle species (Figure 4). However, further research is needed to characterize loggerhead diets in the northeastern GoM and explore their dietary preferences. This would facilitate a comparison between loggerhead and Kemp's ridley turtles to investigate whether the latter forages opportunistically and/or is selecting for less common prey to minimize competition.

This study is one of the first marine turtle studies to include sulfur (δ^34^S) as a third isotopic tracer for analyzing epidermal tissue. However, likely due to the limited spatial extent of the study site, the use of δ^34^S did not contribute substantially more information beyond δ^13^C and δ^15^N, as it has in previous marine consumer studies that sample consumers from a wider geographic range (e.g., Borrell et al., 2021; Bradshaw et al., 2017; García‐Vernet et al., 2021; Guillemin et al., 2022). Nonetheless, the addition of sulfur isotopes here proved valuable by suggesting a micro‐habitat preference by loggerhead turtles, which would not have been observed based on carbon and nitrogen isotopes alone.

The present study did not incorporate data on potential prey items due to the lack of adequate prey samples for isotopic analysis. Our goal instead was to provide an analysis of isotopic niche variation and overlap among sympatric marine turtles within a shared foraging area and establish a foundation for future studies. Future efforts should collect potential prey items and apply stable isotope mixing models to further investigate interspecific differences by prey taxa that may not be possible from this study. It is also worth noting the limitations inherent to isotope‐based metrics of the ecological niche, as there are other axes not considered here (e.g., temporal partitioning). Nonetheless, our isotopic analysis revealed a high degree of trophic and spatial overlap among the three species, which suggests that they use similar resources when inhabiting the same foraging habitat. These results highlight the importance of this area to marine turtles and the need for ongoing conservation efforts, which could include expanding protection zones and maintaining regulations for recreational and commercial fisheries in the region.

## AUTHOR CONTRIBUTIONS

**Savannah Weber:** Conceptualization (equal); data curation (equal); formal analysis (lead); investigation (lead); methodology (equal); visualization (lead); writing – original draft (lead); writing – review and editing (lead). **Joshua A. Cullen:** Conceptualization (equal); formal analysis (equal); investigation (equal); methodology (lead); software (lead); supervision (equal); visualization (equal); writing – original draft (supporting); writing – review and editing (equal). **Mariana M. P. B. Fuentes:** Conceptualization (equal); data curation (lead); formal analysis (supporting); funding acquisition (lead); investigation (equal); methodology (equal); project administration (lead); resources (lead); supervision (lead); visualization (supporting); writing – original draft (supporting); writing – review and editing (lead).

## CONFLICT OF INTEREST STATEMENT

The authors declare that they have no competing interests.

## Supporting information


Appendix S1

## Data Availability

The bulk stable isotope dataset and the R scripts generated during the current study are available on Zenodo: https://doi.org/10.5281/zenodo.8286651.

## References

[ece310741-bib-0001] Arthur, K. E. , Boyle, M. C. , & Limpus, C. J. (2008). Ontogenetic changes in diet and habitat use in green sea turtle (*Chelonia mydas*) life history. Marine Ecology Progress Series, 362, 303–311. 10.3354/meps07440

[ece310741-bib-0002] Barichivich, W. J. (2006). Characterization of a marine turtle aggregation in the Big Bend of Florida. Thesis, University of Florida.

[ece310741-bib-0003] Barichivich, W. J. , Sulak, K. J. , & Carthy, R. R. (1999). Feeding ecology and habitat affinities of Kemp's ridley sea turtles (*Lepidochelys kempi*) in the Big Bend, Florida: Final report. http://aquaticcommons.org/id/eprint/957

[ece310741-bib-0004] Benscoter, A. M. , Smith, B. J. , & Hart, K. M. (2022). Loggerhead marine turtles (*Caretta caretta*) nesting at smaller sizes than expected in the Gulf of Mexico: Implications for turtle behavior, population dynamics, and conservation. Conservation Science and Practice, 4, e581. 10.1111/csp2.581

[ece310741-bib-0005] Bjorndal, K. A. (1985). Nutritional ecology of sea turtles. Copeia, 1985, 736–751. 10.2307/1444767

[ece310741-bib-0006] Bjorndal, K. A. , & Bolten, A. B. (1995). Comparison of length‐frequency analyses for estimation of growth parameters for a population of green turtles. Herpetologica, 51, 160–167.

[ece310741-bib-0007] Bjorndal, K. A. , Bolten, A. B. , Koike, B. , Schroeder, B. A. , Shaver, D. J. , Teas, W. G. , & Witzell, W. N. (2001). Somatic growth function for immature loggerhead sea turtles, *Caretta caretta*, in southeastern U.S. waters. Fishery Bulletin, 99, 240.

[ece310741-bib-0008] Bolten, A. B. (1999). Techniques for measuring sea turtles. Research and management techniques for the conservation of sea turtles (pp. 110–111). IUCN/SSC Marine Turtle Specialist Group.

[ece310741-bib-0009] Borrell, A. , Gazo, M. , Aguilar, A. , Raga, J. A. , Degollada, E. , Gozalbes, P. , & García‐Vernet, R. (2021). Niche partitioning amongst northwestern Mediterranean cetaceans using stable isotopes. Progress in Oceanography, 193, 102559. 10.1016/j.pocean.2021.102559

[ece310741-bib-0010] Bradshaw, P. J. , Broderick, A. C. , Carreras, C. , Inger, R. , Fuller, W. , Snape, R. , Stokes, K. L. , & Godley, B. J. (2017). Satellite tracking and stable isotope analysis highlight differential recruitment among foraging areas in green turtles. Marine Ecology Progress Series, 582, 201–214. 10.3354/meps12297

[ece310741-bib-0011] Burke, V. J. , Standora, E. A. , & Morreale, S. J. (1993). Diet of Juvenile Kemp's Ridley and Loggerhead Sea turtles from long Island, New York. Copeia, 1993, 1176–1180. 10.2307/1447107

[ece310741-bib-0012] Cardona, L. , Campos, P. , Levy, Y. , Demetropoulos, A. , & Margaritoulis, D. (2010). Asynchrony between dietary and nutritional shifts during the ontogeny of green turtles (*Chelonia mydas*) in the Mediterranean. Journal of Experimental Marine Biology and Ecology, 393(1–2), 83–89. 10.1016/j.jembe.2010.07.004

[ece310741-bib-0013] Ceriani, S. A. , Roth, J. D. , Sasso, C. R. , McClellan, C. M. , James, M. C. , Haas, H. L. , Smolowitz, R. J. , Evans, D. R. , Addison, D. S. , Bagley, D. A. , Ehrhart, L. M. , & Weishampel, J. F. (2014). Modeling and mapping isotopic patterns in the Northwest Atlantic derived from loggerhead sea turtles. Ecosphere, 5, art122. 10.1890/ES14-00230.1

[ece310741-bib-0014] Chabot, R. , Welsh, R. , Mott, C. , Guertin, J. , Shamblin, B. , & Witherington, B. (2021). A sea turtle population assessment for Florida's big bend, northeastern Gulf of Mexico. Gulf and Caribbean Research, 32, 19–33. 10.18785/gcr.3201.05

[ece310741-bib-0015] Chan, A. J. , Raoult, V. , Jaine, F. R. A. , Peddemors, V. M. , Broadhurst, M. K. , & Williamson, J. E. (2022). Trophic niche of Australian cownose rays (*Rhinoptera neglecta*) and whitespotted eagle rays (*Aetobatus ocellatus*) along the east coast of Australia. Journal of Fish Biology, 100, 970–978. 10.1111/jfb.15028 35225353 PMC9310580

[ece310741-bib-0016] Chase, J. M. , & Leibold, M. A. (2003). Ecological niches: Linking classical and contemporary approaches. University of Chicago Press.

[ece310741-bib-0017] Chesson, P. (2000). Mechanisms of maintenance of species diversity. Annual Review of Ecology and Systematics, 31, 343–366. 10.1146/annurev.ecolsys.31.1.343

[ece310741-bib-0018] Clyde‐Brockway, C. E. , Heidemeyer, M. , Paladino, F. V. , & Flaherty, E. A. (2022). Diet and foraging niche flexibility in green and hawksbill turtles. Marine Biology, 169, 108. 10.1007/s00227-022-04092-1

[ece310741-bib-0019] Conant, T. A. , Dutton, P. H. , Eguchi, T. , Epperly, S. P. , Fahy, C. C. , Godfrey, M. H. , MacPherson, S. L. , Possardt, E. E. , Schroeder, B. A. , & Seminoff, J. A. (2009). Loggerhead sea turtle (*Caretta caretta*) 2009 status review under the US Endangered Species Act. *Report of the loggerhead biological review Team to the National Marine Fisheries Service* 222, 5‐2.

[ece310741-bib-0020] Connell, J. H. (1961). The influence of interspecific competition and other factors on the distribution of the barnacle *Chthamalus Stellatus* . Ecology, 42, 710–723. 10.2307/1933500

[ece310741-bib-0021] Connolly, R. M. , Guest, M. A. , Melville, A. J. , & Oakes, J. M. (2004). Sulfur stable isotopes separate producers in marine food‐web analysis. Oecologia, 138, 161–167. 10.1007/s00442-003-1415-0 14593525

[ece310741-bib-0022] Devictor, V. , Clavel, J. , Julliard, R. , Lavergne, S. , Mouillot, D. , Thuiller, W. , Venail, P. , Villéger, S. , & Mouquet, N. (2010). Defining and measuring ecological specialization. Journal of Applied Ecology, 47, 15–25. 10.1111/j.1365-2664.2009.01744.x

[ece310741-bib-0023] DiMatteo, A. , Lockhart, G. , & Barco, S. (2022). Habitat models and assessment of habitat partitioning for Kemp's ridley and loggerhead marine turtles foraging in Chesapeake Bay (USA). Endangered Species Research, 47, 91–107. 10.3354/esr01168

[ece310741-bib-0024] Eaton, C. , McMichael, E. , Witherington, B. , Foley, A. , Hardy, R. , & Meylan, A. (2008). In‐water sea turtle monitoring and research in Florida: Review and recommendations. NMFS‐OPR‐38 U.S. Department of Commerce.

[ece310741-bib-0025] Elafri, A. , Belhamra, M. , & Houhamdi, M. (2017). Comparing habitat preferences of a set of waterbird species wintering in coastal wetlands of North Africa: Implication for management. Ekológia (Bratislava), 36, 158–171. 10.1515/eko-2017-0014

[ece310741-bib-0026] FFWCC . (2016). Marine turtle conservation handbook. https://myfwc.com/media/3133/fwc‐mtconservationhandbook.pdf

[ece310741-bib-0027] Figgener, C. , Bernardo, J. , & Plotkin, P. T. (2019). Beyond trophic morphology: Stable isotopes reveal ubiquitous versatility in marine turtle trophic ecology. Biological Reviews, 94, 1947–1973. 10.1111/brv.12543 31338959 PMC6899600

[ece310741-bib-0028] Filippos, L. S. , Taniguchi, S. , Baldassin, P. , Pires, T. , & Montone, R. C. (2021). Persistent organic pollutants in plasma and stable isotopes in red blood cells of *Caretta caretta*, *Chelonia mydas* and *Lepidochelys olivacea* sea turtles that nest in Brazil. Marine Pollution Bulletin, 167, 112283. 10.1016/j.marpolbul.2021.112283 33799149

[ece310741-bib-0029] Foley, A. , Singel, K. , Dutton, P. , Summers, T. , Redlow, A. , & Lessman, J. (2007). Characteristics of a green turtle (*Chelonia mydas*) assemblage in northwestern Florida determined during a hypothermic stunning event. Gulf of Mexico Science, 25, 131–143. 10.18785/goms.2502.04

[ece310741-bib-0030] Fuentes, M. M. P. B. , Lawler, I. R. , Gyuris, E. , Fuentes, M. M. P. B. , Lawler, I. R. , & Gyuris, E. (2006). Dietary preferences of juvenile green turtles (*Chelonia mydas*) on a tropical reef flat. Wildlife Research, 33, 671–678. 10.1071/WR05081

[ece310741-bib-0031] Fujisaki, I. , Hart, K. , Bucklin, D. , Iverson, A. , Rubio, C. , Lamont, M. , Gonzales Diaz Miron, R. , Burchfield, P. , Peña, J. , & Shaver, D. (2020). Predicting multi‐species foraging hotspots for marine turtles in the Gulf of Mexico. Endangered Species Research, 43, 253–266. 10.3354/esr01059

[ece310741-bib-0032] Gama, L. R. , Fuentes, M. M. P. B. , Trevizani, T. H. , Pellizzari, F. , Lemons, G. E. , Seminoff, J. A. , & Domit, C. (2021). Trophic ecology of juvenile green turtles in the Southwestern Atlantic Ocean: Insights from stable isotope analysis and niche modelling. Marine Ecology Progress Series, 678, 139–152. 10.3354/meps13868

[ece310741-bib-0033] García‐Vernet, R. , Borrell, A. , Víkingsson, G. , Halldórsson, S. D. , & Aguilar, A. (2021). Ecological niche partitioning between baleen whales inhabiting Icelandic waters. Progress in Oceanography, 199, 102690. 10.1016/j.pocean.2021.102690

[ece310741-bib-0034] Gardner, S. C. , Fitzgerald, S. L. , Vargas, B. A. , & Rodríguez, L. M. (2006). Heavy metal accumulation in four species of sea turtles from the Baja California peninsula, Mexico. Biometals, 19, 91–99. 10.1007/s10534-005-8660-0 16502335

[ece310741-bib-0035] Godley, B. , Thompson, D. , Waldron, S. , & Furness, R. (1998). The trophic status of marine turtles as determined by stable isotope analysis. Marine Ecology Progress Series, 166, 277–284. 10.3354/meps166277

[ece310741-bib-0036] Guillemin, T. A. , Pepperell, J. G. , Gaston, T. , & Williamson, J. E. (2022). Deciphering the trophic ecology of three Marlin species using stable isotope analysis in temperate waters off southeastern Australia. Frontiers in Marine Science, 9, 1–11. 10.3389/fmars.2022.795436 35450130

[ece310741-bib-0037] Hart, K. M. , Iverson, A. R. , Fujisaki, I. , Lamont, M. M. , Bucklin, D. , & Shaver, D. J. (2018). Sympatry or syntopy? Investigating drivers of distribution and co‐occurrence for two imperiled sea turtle species in Gulf of Mexico neritic waters. Ecology and Evolution, 8, 12656–12669. 10.1002/ece3.4691 30619571 PMC6308884

[ece310741-bib-0038] Haywood, J. C. , Fuller, W. J. , Godley, B. J. , Shutler, J. D. , Widdicombe, S. , & Broderick, A. C. (2019). Global review and inventory: How stable isotopes are helping us understand ecology and inform conservation of marine turtles. Marine Ecology Progress Series, 613, 217–245. 10.3354/meps12889

[ece310741-bib-0039] Herren, R. M. (2018). Sea turtle abundance and demographic measurements in a marine protected area in the Florida Keys, USA. Herpetological Conservation and Biology, 13, 224–239.

[ece310741-bib-0040] HilleRisLambers, J. , Adler, P. B. , Harpole, W. S. , Levine, J. M. , & Mayfield, M. M. (2012). Rethinking community assembly through the lens of coexistence theory. Annual Review of Ecology, Evolution, and Systematics, 43, 227–248. 10.1146/annurev-ecolsys-110411-160411

[ece310741-bib-0041] Hussey, N. E. , MacNeil, M. A. , McMeans, B. C. , Olin, J. A. , Dudley, S. F. J. , Cliff, G. , Wintner, S. P. , Fennessy, S. T. , & Fisk, A. T. (2014). Rescaling the trophic structure of marine food webs. Ecology Letters, 17, 239–250. 10.1111/ele.12226 24308860 PMC3912912

[ece310741-bib-0042] Hutchinson, G. E. (1961). The paradox of the plankton. The American Naturalist, 95, 137–145. 10.1086/282171

[ece310741-bib-0043] Hutchinson, G. E. (1978). An introduction to population ecology. Yale University Press.

[ece310741-bib-0044] Jackson, A. L. , Inger, R. , Parnell, A. C. , & Bearhop, S. (2011). Comparing isotopic niche widths among and within communities: SIBER – Stable isotope Bayesian ellipses in R. Journal of Animal Ecology, 80, 595–602. 10.1111/j.1365-2656.2011.01806.x 21401589

[ece310741-bib-0045] Kent, F. E. A. , Mair, J. M. , Newton, J. , Lindenbaum, C. , Porter, J. S. , & Sanderson, W. G. (2017). Commercially important species associated with horse mussel (*Modiolus modiolus*) biogenic reefs: A priority habitat for nature conservation and fisheries benefits. Marine Pollution Bulletin, 118, 71–78. 10.1016/j.marpolbul.2017.02.051 28222862

[ece310741-bib-0046] Lamont, M. M. , Alday, J. , & Alday, C. (2022). Interspecific interactions among three species of sea turtle using a common resting area. Ecology, 104, e3861. 10.1002/ecy.3861 36062327 PMC10078534

[ece310741-bib-0047] Lamont, M. M. , & Iverson, A. R. (2018). Shared habitat use by juveniles of three sea turtle species. Marine Ecology Progress Series, 606, 187–200. 10.3354/meps12748

[ece310741-bib-0048] Lamont, M. M. , & Johnson, D. (2021). Variation in species composition, size and fitness of two multi‐Species Sea turtle assemblages using different neritic habitats. Frontiers in Marine Science, 7, 1–11. 10.3389/fmars.2020.608740

[ece310741-bib-0049] Laub, B. G. , & Budy, P. (2015). Assessing the likely effectiveness of multispecies management for imperiled desert fishes with niche overlap analysis. Conservation Biology, 29, 1153–1163. 10.1111/cobi.12457 25627117

[ece310741-bib-0050] Limpus, C. J. , & Walter, D. G. (1980). The growth of immature green turtles (*Chelonia mydas*) under natural conditions. Herpetologica, 36, 162–165.

[ece310741-bib-0051] Lysy, M. , Stasko, A. D. , & Swanson, H. K. (2014). *nicheROVER: (niche) (R)egion and niche (over)lap metrics for multidimensional ecological niches* (version 1.1.0).

[ece310741-bib-0052] MacArthur, R. , & Levins, R. (1964). Competition, habitat selection, and character displacement in a patchy environment. Proceedings of the National Academy of Sciences of the United States of America, 51, 1207–1210. 10.1073/pnas.51.6.1207 14215645 PMC300237

[ece310741-bib-0053] Martins, R. F. , Andrades, R. , Nagaoka, S. M. , Martins, A. S. , Longo, L. L. , Ferreira, J. S. , Bastos, K. V. , Joyeux, J.‐C. , & Santos, R. G. (2020). Niche partitioning between sea turtles in waters of a protected tropical Island. Regional Studies in Marine Science, 39, 101439. 10.1016/j.rsma.2020.101439

[ece310741-bib-0054] McGill, B. J. , Enquist, B. J. , Weiher, E. , & Westoby, M. (2006). Rebuilding community ecology from functional traits. Trends in Ecology & Evolution, 4, 178–185. 10.1016/j.tree.2006.02.002 16701083

[ece310741-bib-0055] Melo‐Merino, S. M. , Reyes‐Bonilla, H. , & Lira‐Noriega, A. (2020). Ecological niche models and species distribution models in marine environments: A literature review and spatial analysis of evidence. Ecological Modelling, 415, 108837. 10.1016/j.ecolmodel.2019.108837

[ece310741-bib-0056] Monda, M. J. , & Ratti, J. T. (1988). Niche overlap and habitat use by sympatric duck broods in eastern Washington. The Journal of Wildlife Management, 52, 95–103. 10.2307/3801066

[ece310741-bib-0057] Newsome, S. D. , Martinez del Rio, C. , Bearhop, S. , & Phillips, D. L. (2007). A niche for isotopic ecology. Frontiers in Ecology and the Environment, 5, 429–436. 10.1890/060150.1

[ece310741-bib-0058] Oksanen, S. M. , Niemi, M. , Ahola, M. P. , & Kunnasranta, M. (2015). Identifying foraging habitats of Baltic ringed seals using movement data. Movement Ecology, 3, 33. 10.1186/s40462-015-0058-1 26401285 PMC4580415

[ece310741-bib-0059] Palmer, J. L. , Beton, D. , Çiçek, B. A. , Davey, S. , Duncan, E. M. , Fuller, W. J. , Godley, B. J. , Haywood, J. C. , Hüseyinoğlu, M. F. , Omeyer, L. C. M. , Schneider, M. J. , Snape, R. T. E. , & Broderick, A. C. (2021). Dietary analysis of two sympatric marine turtle species in the eastern Mediterranean. Marine Biology, 168, 94. 10.1007/s00227-021-03895-y

[ece310741-bib-0060] Peterson, B. J. , & Fry, B. (1987). Stable isotopes in ecosystem studies. Annual Review of Ecology and Systematics, 18, 293–320. 10.1146/annurev.es.18.110187.001453

[ece310741-bib-0061] Plotkin, P. T. , Wicksten, M. K. , & Amos, A. F. (1993). Feeding ecology of the loggerhead sea turtle *Caretta caretta* in the Northwestern Gulf of Mexico. Marine Biology, 115, 1–5. 10.1007/BF00349379

[ece310741-bib-0062] Post, D. M. (2002). Using stable isotopes to estimate trophic position: Models, methods, and assumptions. Ecology, 83, 703–718. 10.1890/0012-9658(2002)083[0703:USITET]2.0.CO;2

[ece310741-bib-0063] R Core Team . (2021). R: A language and environment for statistical computing. R Foundation for Statistical Computing.

[ece310741-bib-0064] Radabaugh, K. R. , Hollander, D. J. , & Peebles, E. B. (2013). Seasonal δ13C and δ15N isoscapes of fish populations along a continental shelf trophic gradient. Continental Shelf Research, 68, 112–122. 10.1016/j.csr.2013.08.010

[ece310741-bib-0065] Reich, K. J. , Bjorndal, K. A. , & Bolten, A. B. (2007). The ‘lost years’ of green turtles: Using stable isotopes to study cryptic lifestages. Biology Letters, 3, 712–714. 10.1098/rsbl.2007.0394 17878144 PMC2391226

[ece310741-bib-0066] Reich, K. J. , Bjorndal, K. A. , & Martínez del Rio, C. (2008). Effects of growth and tissue type on the kinetics of 13C and 15N incorporation in a rapidly growing ectotherm. Oecologia, 155, 651–663. 10.1007/s00442-007-0949-y 18188602

[ece310741-bib-0067] Ross, S. T. (1986). Resource partitioning in fish assemblages: A review of field studies. Copeia, 1986, 352–388. 10.2307/1444996

[ece310741-bib-0068] Rossman, S. , Ostrom, P. H. , Gordon, F. , & Zipkin, E. F. (2016). Beyond carbon and nitrogen: Guidelines for estimating three‐dimensional isotopic niche space. Ecology and Evolution, 6, 2405–2413. 10.1002/ece3.2013 27110351 PMC4834325

[ece310741-bib-0069] Sale, P. F. (1974). Overlap in resource use, and interspecific competition. Oecologia, 17, 245–256. 28308169 10.1007/BF00344924

[ece310741-bib-0070] Schmid, J. R. , Bolten, A. B. , Bjorndal, K. A. , Lindberg, W. J. , Percival, H. F. , & Zwick, P. D. (2003). Home range and habitat use by Kemp's Ridley turtles in West‐Central Florida. The Journal of Wildlife Management, 67, 196–206. 10.2307/3803075

[ece310741-bib-0071] Seminoff, J. A. , Komoroske, L. M. , Amorocho, D. , Arauz, R. , Chacón‐Chaverrí, D. , de Paz, N. , Dutton, P. H. , Donoso, M. , Heidemeyer, M. , Hoeffer, G. , Todd Jones, T. , Kelez, S. , Lemons, G. E. , Rguez‐Baron, J. M. , Sampson, L. , Santos Baca, L. , Steiner, T. , Vejar Rubio, M. , Zárate, P. , … Popp, B. N. (2021). Large‐scale patterns of green turtle trophic ecology in the eastern Pacific Ocean. Ecosphere, 12, e03479. 10.1002/ecs2.3479

[ece310741-bib-0072] Servis, J. A. , Lovewell, G. , & Tucker, A. D. (2015). Diet analysis of subadult Kemp's Ridley (*Lepidochelys kempii*) turtles from West‐Central Florida. Chelonian Conservation and Biology, 14, 173–181. 10.2744/CCB-1177.1

[ece310741-bib-0073] Silver‐Gorges, I. , Ceriani, S. A. , & Fuentes, M. M. (2023). Fine‐scale intraspecific niche partitioning in a highly mobile, marine megafauna species: Implications for ecology and conservation. Royal Society Open Science, 10(6), 221529. 37388320 10.1098/rsos.221529 PMC10300683

[ece310741-bib-0074] Silver‐Gorges, I. , Ingels, J. , dos Santos, G. A. P. , Valdes, Y. , Pontes, L. P. , Silva, A. C. , Neres, P. F. , Shantharam, A. , Perry, D. , Richterkessing, A. , Sanchez‐Zarate, S. , Acevedo, L. , Gillis, A. J. , Ceriani, S. A. , & Fuentes, M. M. P. B. (2021). Epibionts reflect spatial and foraging ecology of Gulf of Mexico loggerhead turtles (*Caretta caretta*). Frontiers in Ecology and Evolution, 9, 1–11.

[ece310741-bib-0075] Stewart, J. D. , Rohner, C. A. , Araujo, G. , Avila, J. , Fernando, D. , Forsberg, K. , Ponzo, A. , Rambahiniarison, J. M. , Kurle, C. M. , & Semmens, B. X. (2017). Trophic overlap in mobulid rays: Insights from stable isotope analysis. Marine Ecology Progress Series, 580, 131–151.

[ece310741-bib-0076] Stringell, T. B. , Clerveaux, W. V. , Godley, B. J. , Kent, F. E. A. , Lewis, E. D. G. , Marsh, J. E. , Phillips, Q. , Richardson, P. B. , Sanghera, A. , & Broderick, A. C. (2016). Taxonomic distinctness in the diet of two sympatric marine turtle species. Marine Ecology, 37, 1036–1049. 10.1111/maec.12349

[ece310741-bib-0077] Sullivan, M. J. , & Moncreiff, C. A. (1990). Edaphic algae are an important component of salt marsh food‐webs: Evidence from multiple stable isotope analyses. Marine Ecology Progress Series, 62, 149–159.

[ece310741-bib-0078] Swanson, H. K. , Lysy, M. , Power, M. , Stasko, A. D. , Johnson, J. D. , & Reist, J. D. (2015). A new probabilistic method for quantifying n‐dimensional ecological niches and niche overlap. Ecology, 96, 318–324. 10.1890/14-0235.1 26240852

[ece310741-bib-0079] Tucker, A. , MacDonald, B. , & Seminoff, J. (2014). Foraging site fidelity and stable isotope values of loggerhead turtles tracked in the Gulf of Mexico and Northwest Caribbean. Marine Ecology Progress Series, 502, 267–279. 10.3354/meps10655

[ece310741-bib-0080] Valverde, R. A. , & Holzwart, K. R. (2017). Sea turtles of the Gulf of Mexico. In C. H. Ward (Ed.), Habitats and biota of the Gulf of Mexico: Before the Deepwater horizon oil spill: Volume 2: Fish resources, fisheries, sea turtles, avian resources, marine mammals, diseases and mortalities (pp. 1189–1351). Springer. 10.1007/978-1-4939-3456-0_3

[ece310741-bib-0081] Vander Zanden, H. , Arthur, K. , Bolten, A. , Popp, B. , Lagueux, C. , Harrison, E. , Campbell, C. , & Bjorndal, K. (2013). Trophic ecology of a green turtle breeding population. Marine Ecology Progress Series, 476, 237–249. 10.3354/meps10185

[ece310741-bib-0082] Weber, S. , Ceriani, S. A. , & Fuentes, M. M. (2023). Foraging ecology of Kemp's ridley (*Lepidochelys kempii*) turtles in the northeastern Gulf of Mexico: Insights from stable isotope analysis. Marine Biology, 170(8), 104.

[ece310741-bib-0083] Wildermann, N. , Sasso, C. , Gredzens, C. , & Fuentes, M. M. P. B. (2020). Assessing the effect of recreational scallop harvest on the distribution and behaviour of foraging marine turtles. Oryx, 54, 307–314. 10.1017/S0030605318000182

[ece310741-bib-0084] Wildermann, N. E. , Sasso, C. R. , Stokes, L. W. , Snodgrass, D. , & Fuentes, M. M. P. B. (2019). Habitat use and behavior of multiple species of marine turtles at a foraging area in the northeastern Gulf of Mexico. Frontiers in Marine Science, 6, 155. 10.3389/fmars.2019.00155

[ece310741-bib-0085] Williams, N. C. , Bjorndal, K. A. , Lamont, M. M. , & Carthy, R. R. (2014). Winter diets of immature green turtles (*Chelonia mydas*) on a northern feeding ground: Integrating stomach contents and stable isotope analyses. Estuaries and Coasts, 37, 986–994. 10.1007/s12237-013-9741-x

[ece310741-bib-0086] Witzell, W. N. , & Schmid, J. R. (2005). Diet of immature Kemp's ridley turtles (*Lepidochelys kempi*) from Gullivan Bay, Ten Thousand Islands, Southwest Florida. Bulletin of Marine Science, 77, 191–200.

