# Supplementary material for: Isotopic niche overlap among foraging marine turtle species in the Gulf of Mexico
Source: Ecol Evol. 2023 Nov 28;13(11):e10741. doi: 10.1002/ece3.10741 (PMC10682896; doi:10.1002/ece3.10741)
Supplement: Supplementary file 1 — Appendix S1 [file ECE3-13-e10741-s001.docx]

**Isotopic niche overlap among foraging marine turtle species in the Gulf of Mexico**

Savannah Weber^1^, Joshua A. Cullen^1^, Mariana M. P. B. Fuentes^1^

*^1^Department of Earth, Ocean, and Atmospheric Science, Florida State University, Tallahassee, Florida, United States*

**Supporting Information**

**Table S1**. Summary of isotope analyses, analytical precision values, and sample sizes conducted at the three stable isotope laboratories.

| **Lab** | **Isotopes Analyzed** | **Analytical Precision** | **Sample Size** |
| --- | --- | --- | --- |
| MBL | δ^13^C, δ^15^N, δ^34^S | δ^13^C: +/- 0.1 ‰  δ^15^N: +/- 0.1 ‰  δ^34^S: +/- 0.35 ‰ | n=32 |
| FWRI USF  + MBL | δ^13^C, δ^15^N  + δ^34^S | δ^13^C: +/- 0.17 ‰  δ^15^N: +/- 0.25 ‰  δ^34^S: +/- 0.35 ‰ | n=189* |
| WSU | δ^13^C, δ^15^N, δ^34^S | δ^13^C: +/- 0.5 ‰  δ^15^N: +/- 0.5 ‰  δ^34^S: +/- 0.5 ‰ | n=27 |
| *Total* | δ^13^C, δ^15^N, δ^34^S |  | n=248 |

*Note*. *For these samples, δ^13^C and δ^15^N analyses were conducted at FWRI USF, δ^34^S analysis was conducted at MBL. Abbreviations: FWRI USF = Fish and Wildlife Research Institute of the Florida Fish and Wildlife Conservation Commission and Marine Environmental Chemistry Laboratory at the University of South Florida College of Marine Science; MBL = Marine Biological Laboratory Stable Isotope Laboratory (Woods Hole, Massachusetts); WSU = Washington State University Stable Isotope Core Laboratory.

**
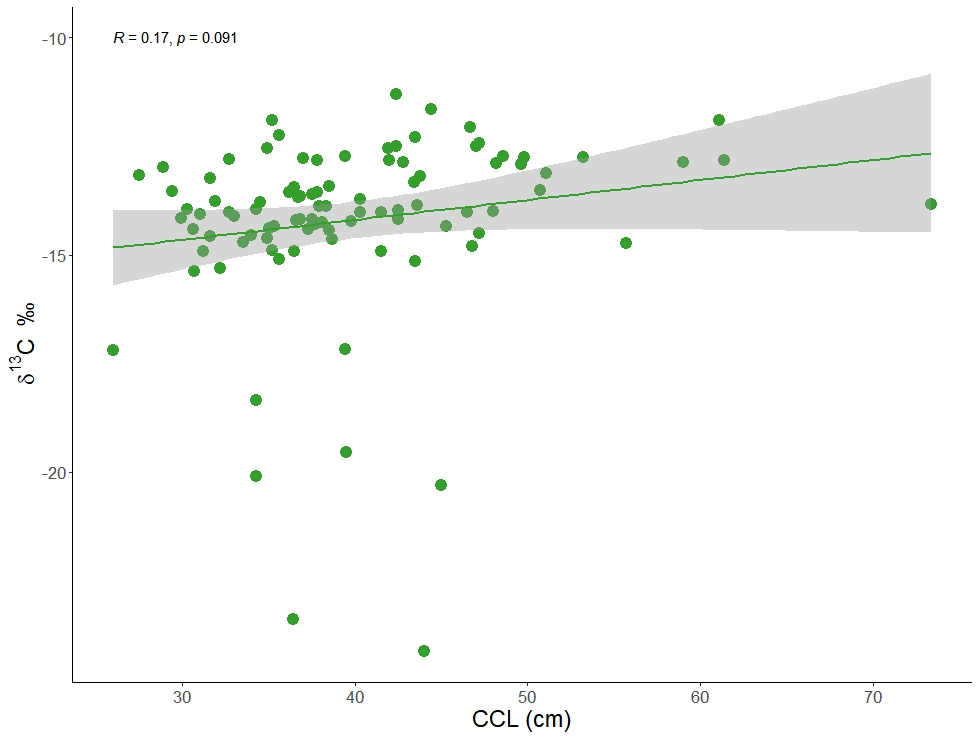
 Figure S1.** Plot of δ^13^C and CCL (curved carapace length) for green turtles (p=0.091, 95% CI[-

0.007, 0.099]). Grey shading represents the 95% confidence interval.


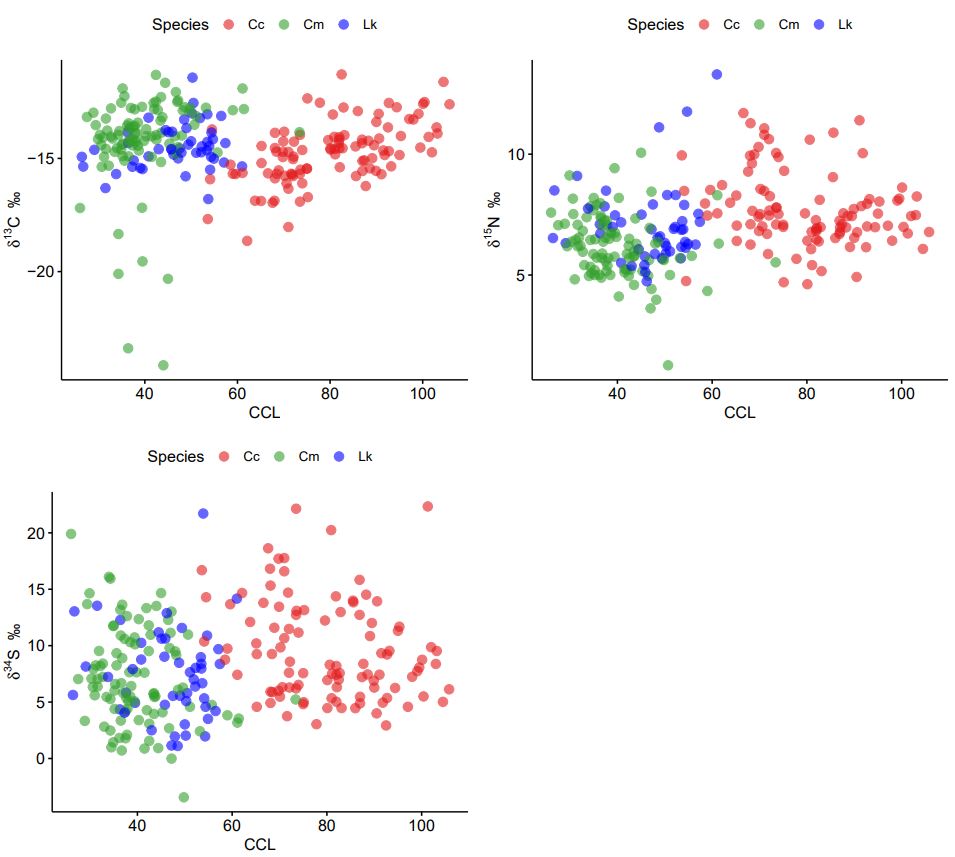


**Figure S2.** Scatter plots of CCL (curved carapace length) and δ^13^C, δ^15^N, and δ^34^S for loggerhead, green, and Kemp’s ridley turtles.


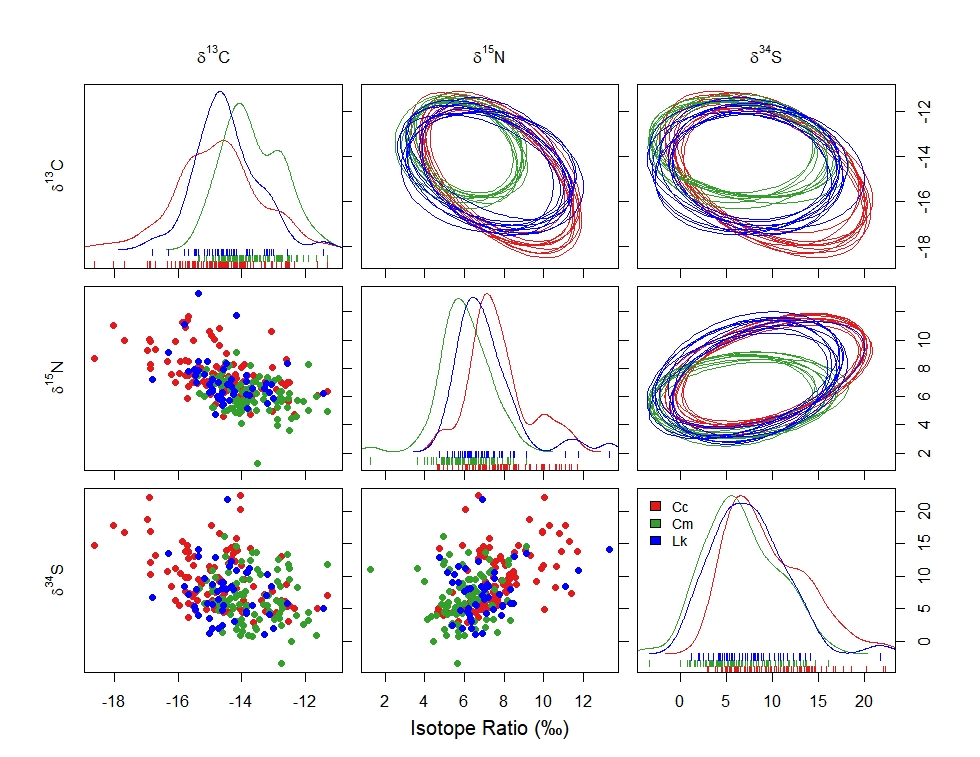


**Figure S3.** NicheROVER plots for loggerhead (Cc), green (Cm) (with the 8 potential outliers omitted), and Kemp’s ridley (Lk) turtles sampled off the coast of Crystal River, Florida, USA between 2016 and 2022. Top-right: Ten random samples of two-dimensional ellipses from the posterior distribution (95% probability region) for each pair of isotope ratios. Diagonal: One-dimensional density plots showing distribution of isotope values with rug plots to show individual values. Bottom-left: Scatterplots of raw data for each pair of isotopes.
